# Comprehensive profiling of arsenosugars in algae using UHPLC-HRMS and UHPLC-IMS-Q-TOF

**DOI:** 10.1007/s00216-026-06454-w

**Published:** 2026-03-27

**Authors:** Alba Morales-Rodríguez, Àngels Sahuquillo, José Fermín López-Sánchez, Dolores Barrón, Encarnación Moyano

**Affiliations:** 1 Departament d’Enginyeria Química i Química Analítica, Universitat de Barcelona, Martí i Franquès, 1-11, 08028 Barcelona, Spain; 2 Departament de Nutrició, Ciències de l’Alimentació i Gastronomia, Campus de l’Alimentació de Torribera, Universitat de Barcelona, Avda. Prat de la Riba, 171, 08921, Sta. Coloma de Gramenet, Barcelona, Spain; 3 Institut de Recerca en Nutrició i Seguretat Alimentària, Universitat de Barcelona (INSA-UB, Recognized as a Maria de Maeztu Unit of Excellence grant (CEX2021 001234-M)), Barcelona, Spain; 4 Institut de Recerca de l’Aigua, Universitat de Barcelona (IdRA-UB), Barcelona, Spain

**Keywords:** Arsenosugars, Algae, HILIC, HRMS, Ion mobility

## Abstract

Arsenic is a ubiquitous environmental contaminant, and its toxicological impact is highly dependent on its speciation. In marine edible algae, increasingly consumed for their nutritional value, arsenosugars (As-Sugars) constitute the major arsenic forms. Despite their generally low toxicity, their uncertain long-term effects make accurate identification essential. We developed an analytical workflow combining ultra-high-performance liquid chromatography coupled to high-resolution mass spectrometry (UHPLC-HRMS). Both Q-Orbitrap and IMS-Q-TOF systems were employed, operating in positive and negative electrospray ionization (ESI) modes. Using a zwitterionic hydrophilic interaction liquid chromatography (HILIC-Z) column, we achieved effective separation of the target compounds and subsequently performed detailed HRMS and MS/MS analyses. This approach enabled the structural annotation of four key As-Sugars (Gly-Sug, PO_4_-Sug, SO_3_-Sug, and SO_4_-Sug) on the basis of accurate mass measurements and diagnostic fragmentation patterns. Notably, we report for the first time the collision cross-section (CCS) values of these As-Sugars, providing an additional orthogonal parameter that strengthens their confirmation.

**Graphical abstract:**

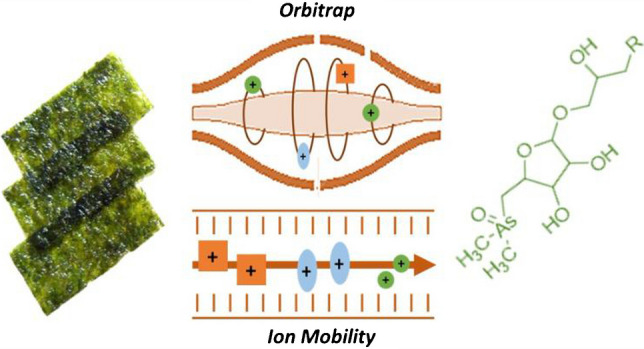

**Supplementary Information:**

The online version contains supplementary material available at 10.1007/s00216-026-06454-w.

## Introduction

Arsenic is a widespread environmental contaminant originating from both natural and anthropogenic sources. Due to its toxicity even at low exposure levels, it is recognized as a significant public health concern. However, quantifying total arsenic in samples is insufficient for assessing toxicological risk, as toxicity depends strongly on the specific chemical species present [1, 2].

Among arsenic compounds, arsenosugars (As-Sugars), organically bound species derived from ribose, are receiving growing attention. They are the predominant arsenic forms in algae [3–5], which are becoming more common in the human diet because of their nutritional value, including high levels of plant-based proteins, minerals, vitamins, and bioactive compounds with potential health benefits [6, 7]. Although As-Sugars are not considered acutely toxic, concerns have been raised about possible mild chronic effects, increasing the need for reliable exposure assessment in populations with high algal consumption [7, 8].

Despite their relevance, current data on the toxicity of As-Sugars remain limited and often inconsistent, largely because outcomes differ depending on whether only the parent compound or also their metabolic products, particularly DMA, are considered. While parent As-Sugars generally show low acute toxicity, their metabolites may raise greater toxicological concern. Reflecting this uncertainty, the European Food Safety Authority (EFSA) has explicitly highlighted the insufficient data on the occurrence, metabolism, and toxicity of As-Sugars and has called for additional information studies to strengthen risk assessment for complex organoarsenic species [9].

Several analytical strategies have been developed for arsenic speciation in marine algae. The first structural characterization of dimethylated As-Sugars was achieved by preparative isolation though successive liquid chromatography steps followed by nuclear magnetic resonance (NMR) spectroscopy [10]. Currently, high-performance liquid chromatography (HPLC) coupled to inductively coupled plasma mass spectrometry (ICP-MS) is the most widely used technique for quantitative arsenic speciation [5, 11], offering diverse stationary phases, high resolution, low detection limits, and robustness against matrix effects [10, 12]. However, some analytical challenges complicate their study, as the structural similarity among As-Sugars species and the lack of commercially available standards hinder unambiguous molecular-level identification by HPLC-MS. HPLC-ICP-MS also has important limitations, such as the requirement for complete chromatographic separation, the lack of molecular structural information, and the need of retention time matching with authentic standards for compound identification and accurate species-specific quantification [13]. Although simultaneously coupling with ESI-MS is feasible when using MS-compatible volatile buffers (e.g. ammonium carbonate or ammonium formate), structural elucidation ultimately depends on the molecular information provided by the ESI-MS rather than on the elemental-specific signal measured by ICP-MS. As a result, arsenic species without commercial standards remain unidentified, and potential co-elution or retention times shifts, particularly in complex biological matrices, must also be considered [12, 14].

These limitations highlight the need for more complementary, more selective detection strategies that provide structural information beyond the capabilities of ICP-MS alone [10]. Although these methods may lack ICP-MS’s quantitative performance and are not always straightforward to couple, they still offer valuable qualitative insights for arsenic species identification. LC-ESI-HRMS, for instance, enables effective structural characterization, including the unambiguous identification of As-Sugars in algae extracts [14–16]. However, because ion suppression is intrinsic to ESI and persists even with HILIC or ion mobility approaches, ESI-MS remains largely qualitative, particularly in the absence of commercial standards [10, 17].

Although the four major As-Sugars commonly found in algae (Gly-Sug, PO_4_-Sug, SO_3_-Sug, and SO_4_-Sug) were first identified several decades ago using low-resolution mass spectrometric approaches such as FAB-MS and early ESI-MS [18–20], their characterization was largely limited by the analytical capabilities available at the time. Advances in UHPLC-HRMS now enable accurate-mass measurements, isotopic fine structure analysis, improved chromatographic resolution, and high-quality MS/MS spectra, capabilities that were not accessible in the earlier foundational studies. In this work, we develop an UHPLC-ESI-HRMS specifically designed for the structural elucidation of As-Sugars in complex algal matrices, providing a modern high-confidence characterization workflow that complements and extends historical As-Sugar data.

## Experimental procedure

### Reagents and materials

LC-MS grade solvents, including water and acetonitrile (ACN) (Honeywell, USA), were used for the preparation of mobile phases. Analytical grade reagents such as ammonium acetate 98% (PanReac, Spain), ammonium formate 99.9% (Sigma-Aldrich-Merck, Germany), ammonium dihydrogenphosphate 99.99% (Merck, Germany), aqueous ammonia solution 25% (Merck, Germany), pyridine (Merck, Germany), and formic acid 98% (PanReac, Spain) were also used as additives for the mobile phases.

### Preparation of HPLC-ICP-MS working solutions

A solution of As (III) with a certified concentration of 1002 ± 4 mg·L^−1^ (Inorganic Ventures) and a solution of As (V) with a certified concentration of 1002 ± 4 mg·L^−1^ (Inorganic Ventures), both traceable to the National Institute of Standards and Technology (NIST), was used as stock standards for inorganic arsenic species.

Other standard solutions (500 mg As·L^−1^) were prepared in aqueous solutions from the following compounds: methylarsonic acid (MMA) from (CH_3_)AsO(ONa)_2_·6H_2_O (Carlo Erba), dimethylarsinic acid (DMA) from (CH_3_)_2_AsNaO_2_·3H_2_O (Fluka), arsenobetaine (AB) from (CH_3_)_3_AsCH_2_COO (Argus Chemicals SRL), arsenocholine (AC) from (CH_3_)_3_AsCH_2_OHBr (Argus Chemicals SRL), and trimethylarsine oxide (TMAO) from (CH_3_)_3_AsO (Argus Chemicals SRL). All stock solutions were stored at 4 °C in polyethylene bottles, and working dilutions were freshly prepared daily for analysis.

### Instrumentation and apparatus

An end-over-end shaker and a HettichZentrifugen 460R centrifuge (Tuttlingen, Germany) were used for the extraction of arsenic species. A Telstar Lyoquest 80 lyophilizer (Tokyo, Japan) was employed to prepare the samples for injection.

The pH of the solutions was measured using a CRISON 2002 potentiometer (± 0.1 mV) (Barcelona, Spain) equipped with a CRISON 5203 combined pH electrode from Orion Research (Boston, MA, USA). An analytical balance with a precision ± 0.1 mg was also used.

#### HPLC-ICP-MS

For the determination of arsenic species, a PerkinElmer NexION 350D inductively coupled plasma mass spectrometer (ICP-MS) with a PFA-ST MicroFlow concentric nebulizer (PerkinElmer, Waltham, MA, USA) was used. Sample introduction was performed directly via the quaternary pump of an Agilent 1100 series HPLC system (Agilent Technologies, Palo Alto, CA, USA). Arsenic quantification was based on ion intensity at *m/z* 75 (^75^As). To detect potential chloride-based interference (^40^Ar^35^Cl) at *m/z* 75, ion signals at *m/z* 35 (^35^Cl) and *m/z* 77 (^40^Ar^37^Cl) were also monitored.

Table S1 summarizes the chromatographic systems and analytical columns used. Quantification was carried out using external calibration curves, with standards selected based on the nearest eluting arsenic species.

#### UHPLC-ESI-Q-Orbitrap-MS

Chromatographic separation was performed using an ultra-high-performance liquid chromatography (UHPLC) system comprising an Accela 1250 pump, Accela autosampler, and column oven (Thermo Fisher Scientific, Waltham, MA, USA). The analytical column used was an InfinityLab Poroshell 120 HILIC-Z (100 mm × 2.1 mm, 2.7 µm, Agilent, Palo Alto, CA, USA), fitted with an ACQUITY column in-line filter (Waters, Milford, MA, USA).

Chromatographic separation was carried out under isocratic elution using a mobile phase consisting of acetonitrile and 10 mM ammonium acetate (ACN: 10 mM NH_4_CH_3_COO, 75:25, *v/v*). The flow rate was set to 200 µL·min^−1^, with an injection volume of 10 µL, and the column temperature maintained at 40 °C.

The UHPLC system was coupled to a Q-Exactive mass spectrometer (Thermo Fisher Scientific, Waltham, MA, USA) equipped with a heated electrospray ionization source (H-ESI II) and a quadrupole-Orbitrap (Q-Orbitrap) mass analyzer. The operating parameters in both positive and negative ionization modes were as follows: spray voltage of 2.5 kV (both polarities), S-lens RF level of 60 V, vaporizer temperature of 350 °C, and ion transfer tube temperature of 250 °C. Nitrogen (Linde, Spain) was used as sheath, auxiliary and sweep gas at flow rates of 40, 20, and 0 arbitrary units (a.u.), respectively.

The Q-Orbitrap operated in a two-event acquisition mode: (1) a full MS scan at a mass resolution of 70,000 full width at half maximum (FWHM) over an *m/z* range of 60–500; and (2) all-ion fragmentation (AIF) with stepped normalized collision energies (NCE) of 20, 30, and 40. Automatic gain control (AGC) was set to 10^−6^, with a maximum injection time of 100 ms.

#### UHPLC-ESI-IMS-Q-TOF-MS

Chromatographic separation was performed using an Agilent 1290 Infinity UHPLC system (Agilent Technologies, Palo Alto, CA, USA). The analytical column and chromatographic conditions used were identical to those described in the “UHPLC-ESI-Q-Orbitrap-MS” section.

The UHPLC system was coupled to a Q-TOF mass spectrometer equipped with an ion mobility cell (IMS-Q-TOF) (Agilent, Palo Alto, CA, USA), using a heated electrospray ionization source (Dual AJS ESI).

Ion mobility separation was achieved via drift tube ion mobility spectrometry (DTIMS). Prior to analysis, the system was calibrated using the Agilent Technologies ESI tune mix to obtain reproducible collision cross-section (CCS) values estimated from the measured drift times. While this calibration does not provide absolute CCS values for non-globular molecules such as As-Sugars, the resulting CCS measurements serve as reliable, comparative parameters supporting structural characterization.

Analyses were initially conducted in positive ionization mode, followed by negative ionization mode. In both cases, data acquisition was performed over an *m/z* range of 100–1700. The ESI source parameters were as follows: gas temperature, 300 °C; drying gas flow rate, 5 L·min^−1^; nebulizer, 35 psi; sheath gas temperature, 350 °C; sheath gas flow rate, 11 L·min^−1^; capillary voltage, 3500 V; and nozzle voltage, 1000 V.

For ion mobility (IM) measurements, the following settings were applied: frame rate, 1 frame·s^−1^; IM transient rate, 16 transients·frame^−1^; maximum drift time, 60 ms; trap fill time, 5000 μs; trap release time, 150 μs; drift tube entrance voltage, 1500 V; drift tube exit voltage, 250 V; rear funnel entrance voltage, 240 V and rear funnel exit voltage, 43 V. Additionally, targeted MS/MS spectra were recorded at 20, 30, and 40 eV.

### Samples

Twelve algae samples were selected from a pool previously analysed in a prior study [7] and were classified into three major taxonomic groups. The Phaeophyta (brown seaweeds) group included *Fucus vesiculosus* (Fucus S01), *Hizikia fusiformis* (Hijiki S03), *Laminaria ochroleuca* (Kombu S07, Kombu S08), *Himanthalia elongata* (Sea spaghetti S10), and *Undaria pinnatifida* (Wakame S15). The Rhodophyta (red seaweeds) group was represented by *Ulva lactuca* (Sea lettuce S16). The Chlorophyta (green seaweeds) group included *Chondrus crispus* (Irish moss S19), *Palmaria palmata* (Dulse S21), and *Porphyra umbilicalis* (Nori S23, Nori S24, Nori S25).

### Sample preparation

Sample preparation and treatment were performed according to the protocol outlined in the referenced publication [7]. Aqueous extracts of each algae species were prepared by weighing 0.25 g of dried algae powder into 15 mL polypropylene tubes, followed by the addition of 10 mL of doubly deionized water. The samples were extracted using an end-over-end shaker at 30 rpm for 16 h at room temperature. After extraction, the suspensions were centrifuged at 3000 rpm for 20 min, and the resulting supernatants were filtered through 0.45 µm nylon filters. The filtered extracts were stored at 4 °C.

For UHPLC-ESI-HRMS analyses using the HILIC column, aliquots of 3 mL of each extract were freeze-dried and subsequently reconstituted in 3 mL of a solvent mixture consisting of acetonitrile and 10 mM ammonium acetate (ACN:NH_4_CH_3_COO, 75:25, *v/v*). Prior to injection, all samples were filtered through 0.20 µm nylon filters. ICP-MS samples were processed separately using ICP-compatible conditions.

### Data analysis

Syngistix for ICP-MS software (PerkinElmer) was used to control and monitor the HPLC-ICP-MS system, while OpenChrom software was employed for data processing.

For UHPLC-HRMS analyses, Xcalibur v2.1 software (Thermo Fisher Scientific) was used to operate the UHPLC-ESI-Q-Orbitrap-MS system and to acquire and process the data. In the case of UHPLC-ESI-IMS-Q-TOF-MS analyses, system control and data acquisition were performed using MassHunter Workstation software (Agilent Technologies), while data processing, including the calculation of CCS values, was carried out using the IM-MS Browser software (Agilent Technologies).

## Results and discussion

### Chromatographic separation

The four As-Sugars investigated in this study (Gly-Sug, PO_4_-Sug, SO_3_-Sug, and SO_4_-Sug) were characterized based on their known chemical structures and physicochemical properties (pK_a_ and log P_o/w_), summarized in Table 1. The pKa and log P_o/w_ values were estimated using cheminformatics prediction tools (ACD/Labs and ChemDraw). These parameters are included to assist in interpreting their chromatographic and ionization behaviour in the UHPL-ESI-HRMS method developed in this work.

**Table 1:**
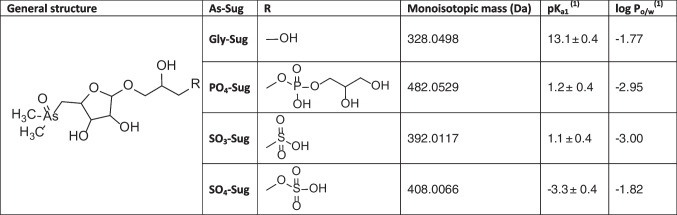
Structure and properties of As-Sugars

^(1)^pK_a_ and log P_o/w_ values were predicted computationally using ACD/Lab and ChemDraw software

Among available chromatographic strategies, Ion Exchange Chromatography (IEX) and Hydrophilic Interaction Chromatography (HILIC) are the most suitable for the separation of As-Sugars. Gly-Sug is typically separated by cation exchange chromatography (CEX), whereas PO_4_-Sug, SO_3_-Sug, and SO_4_-Sug require anion exchange chromatography (AEX). Although IEX-ICP-MS has long been applied for analysis of As-Sugars [21], recent applications [22] have continued to refine and validate this approach.

In this study, we used IEX-ICP-MS for quantitative determination of As-Sugars [22] and evaluated a more ESI-MS-friendly alternative for UHPLC-ESI-HRMS analysis. The sulfoalkylbetaine stationary phase of HILIC-Z provides zwitterionic, mix-mode retention, allowing all four As-Sugars to be separated in a single run while maintaining full compatibility with HRMS. Although complete baseline separation was not achieved under our conditions, HILIC provided effective retention and partial separation, which, combined with HRMS selectivity, enables unambiguous identification despite partial co-elution.

**Fig. 1:**
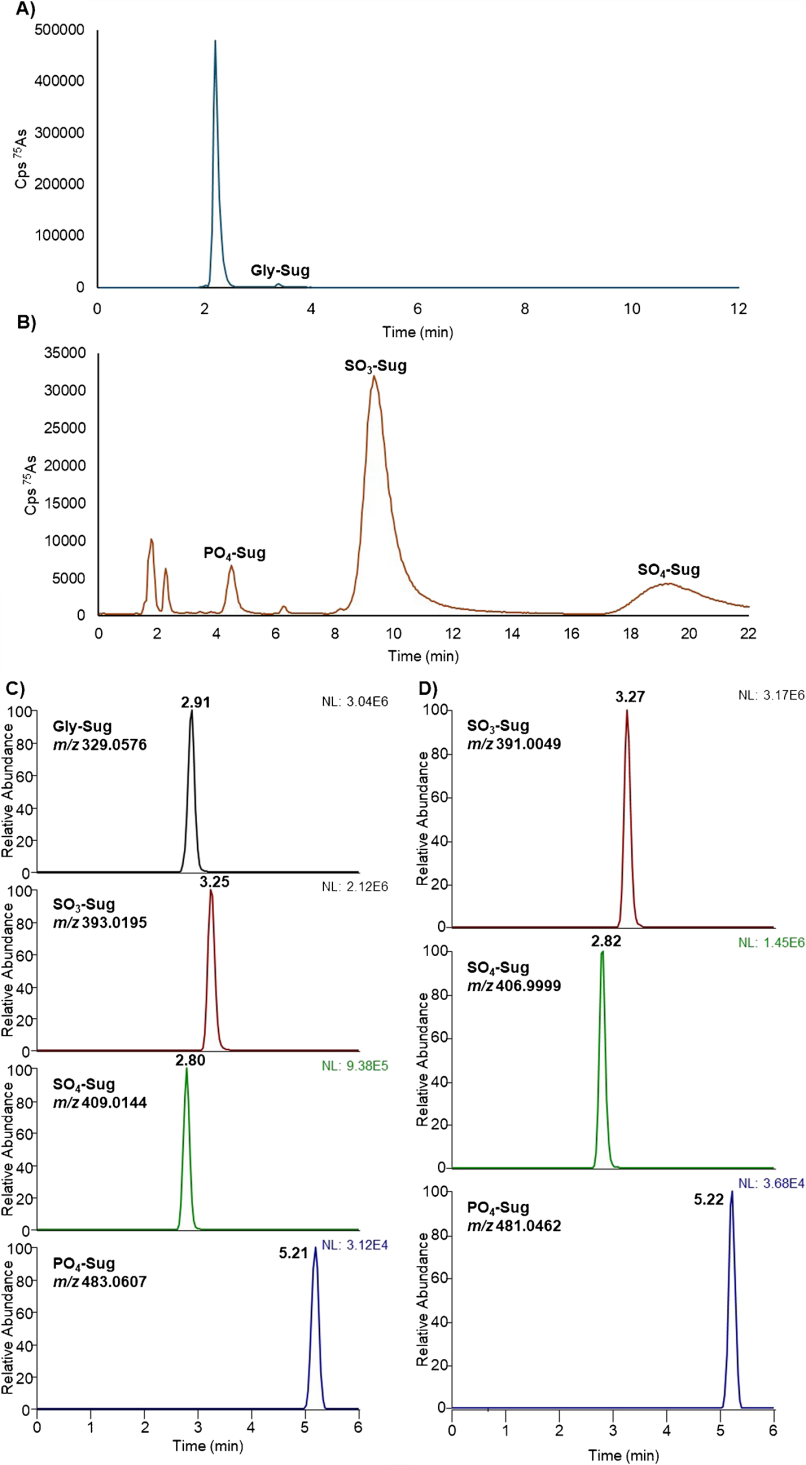
Chromatograms illustrating the separation of As-Sugars in a Fucus extract using three chromatographic methods: **A** cation exchange chromatography (CEX), **B** anion exchange chromatography (AEX), and **C**, **D** hydrophilic interaction chromatography with a Zwitterionic stationary phase (HILIC-Z) under both **C** positive and **D** negative ionization modes

IEX chromatograms for a *Fucus* extract (Fig. 1A, B) showed the expected elution order [7]. Gly-Sug was retained under acidic CEX conditions (20 mM pyridine, pH 2.6), while the AEX method separates the anionic species using NH_4_H_2_PO_4_ buffers (pH 5.8). Increasing the pH up to 8 alters the distribution of phosphate species toward more highly charged forms, producing a corresponding change in ionic strength and affecting analyte retention.

Optimal separation of As-Sugars using HILIC-Z column was achieved under isocratic conditions with acetonitrile and 10 mM NH_4_CH_3_COO (75:25, *v/v*) (Fig. 1C, D). While the chromatographic resolution was modest, the HILIC-Z stationary phase provided sufficient retention and selectivity to allow all four As-Sugars to be distinguished to a degree compatible with confident UHPLC-ESI-HRMS identification. Several retention mechanisms contribute to the observed chromatographic behaviour: electrostatic repulsion led to early elution of the anionic As-Sugars, while partitioning into the water-rich layer on the stationary phase increased retention of more hydrophilic species. Gly-Sug showed slightly longer retention due to cation-anion interactions. Although PO_4_-Sug and SO_3_-Sug exhibit similar hydrophilicity (similar log P_o_/w values), the slightly stronger retention of PO_4_-Sug may arise from structural differences between the two molecules. The bulkier substituent attached to PO_4_-Sug modifies the local charge distribution around the anionic group and increases the potential for secondary interactions (e.g. hydrogen bonding or weak hydrophobic contacts) with the zwitterionic stationary phase, resulting in slightly longer retention.

### Mass spectrometry

In ICP-MS, As-Sugars are atomized in the plasma and detected as arsenic ions at *m/z* 75 with high sensitivity and minimal isotopic interferences. Potential chloride-based polyatomic interferences (e.g. ^40^Ar^35^Cl) were monitored at *m/z* 35 (^35^Cl) and 77 (^40^Ar^37^Cl), but none was detected. However, ICP-MS cannot provide structural information on the organic moieties of As-Sugars.

In contrast, the soft ionization of ESI preserves the molecular integrity and its coupling with HRMS provides accurate-mass and elemental-composition data, together with characteristic MS/MS fragments that support the identification of known As-Sugars. Although HRMS does not provide full structural elucidation in the same way as NMR, it offers sufficiently detailed molecular information for confirming the identity of As-Sugar at the low concentrations present in algal extracts. Both positive and negative modes yielded exclusively singly charged ions, formed by protonation [M+H]^+^ and deprotonation [M-H]^-^. All As-Sugars followed this pattern except Gly-Sug, which produced only [M+H]^+^. Protonation most likely occurs at the As-O site, while deprotonation occurs at sulfonic, sulphuric or phosphate groups (Table 1). Although adduct ions such as [M+Na]^+^ are commonly observed in ESI, none was detected under our optimized conditions. Likewise, no in-source fragments were observed, which favoured the exclusive formation of the protonate and deprotonated species.

**Fig. 2:**
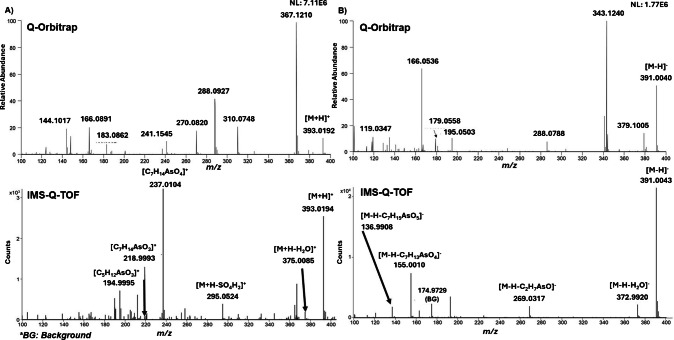
All-ion fragmentation (AIF) mass spectra acquired in **A** positive and **B** negative ionization modes. The upper spectra were recorded using a Q-Orbitrap system, whereas the lower spectra were obtained with an IMS-Q-TOF system

Based on data from a previous study [7], samples with the highest concentrations of As-Sugars were selected for fragmentation analysis (Fucus S01 and Nori S24). AIF experiments were performed on both Q-Orbitrap (HCD) and IMS-Q-TOF (CID) platforms (Fig. 2). Although the fragmentation pathways of As-Sugars have been described previously [23], this comparison was carried out to evaluate the performance of both platforms for structural confirmation in complex algal matrices. Figure 2 shows the spectra obtained for SO_3_-Sug using both platforms. As can be seen, the IMS-Q-TOF generated cleaner AIF spectra due to ion mobility separation prior to CID, which reduces co-fragmentation of matrix ions. For this reason, IMS-Q-TOF AIF spectra were selected for subsequent structural interpretation.

**Fig. 3:**
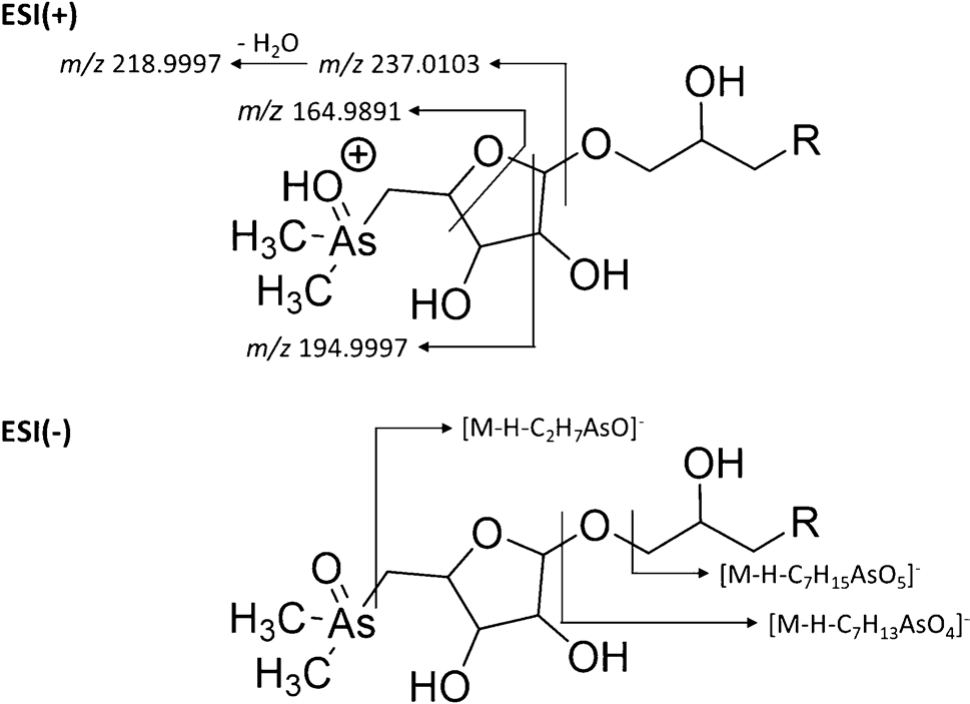
Proposed fragmentation pathways under positive and negative ionization modes

Although the fragmentation behaviour of As-Sugars has been described previously, Fig. 3 summarizes the fragments observed under the specific AIF conditions used in this study, and the ion assignment along with their relative intensities, which depend strongly on the dissociation mechanism (HCD vs CID), applied energies, and instrument settings, are included in the “Experimental procedure” section. The diagnostic ions identified here represent the most consistently observed fragments across our experiments and were used to support the identification of As-Sugars in the algal extracts analysed. In positive mode, fragmentation was dominated by water loss and C-O bond cleavage, generating characteristic arsenic-containing ions such as [C_7_H_14_AsO_4_]^+^ (*m/z* 237.0103), consistently detected in all As-Sugars and therefore an excellent diagnostic marker. Other common fragments appeared at lower intensities or were compound-dependent. Functional group-specific neutral losses were also observed: PO_4_-Sug and SO_4_-Sug showed P-O and S-O bond cleavages, whereas SO_3_-Sug followed a different C-S cleavage pathway with water loss.

In negative mode, deprotonation at acidic functional groups produced two main fragment types: (i) ions resulting from C-O bond cleavages, with the charge remaining on the acidic site (Fig. 3), and (ii) ions derived from specific fragmentation of the functional group itself (Table S2). The common loss C_7_H_13_AsO_4_ occurred in all As-Sugars, whereas losses such as C_2_H_7_AsO, C_7_H_15_AsO_5_, and H_2_O were unique to sulfonate and sulfate analogues. PO_4_-Sug again showed P-O cleavage, consistent with its positive-mode behaviour. Most fragment ions showed mass errors below 5 ppm, with slightly larger deviations for very low abundance low *m/z* fragments.

**Fig. 4:**
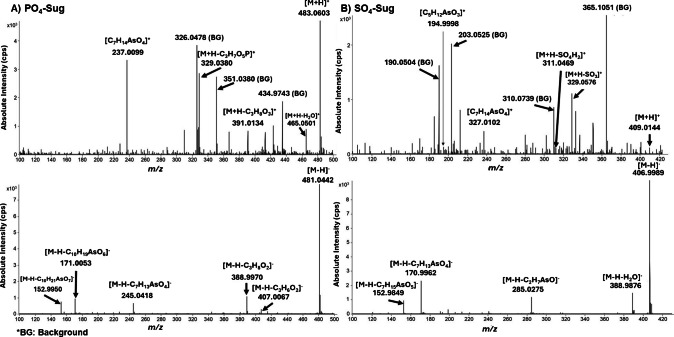
All-ion fragmentation (AIF) mass spectra acquired with the IMS-Q-TOF system for **A** PO_4_-Sug and **B** SO_4_-Sug. The upper spectra correspond to positive ionization mode, and the lower spectra correspond to negative ionization mode

AIF spectra acquired for PO_4_-Sug and SO_4_-Sug in both ionization modes using IMS-Q-TOF (Fig. 4; Fig. S1) show the common fragments and group-specific neutral losses associated with the phosphate and sulfate moieties.

Previous studies reported only low-resolution ion trap MS^2^ (direct fragmentation) or MS^n^ (multi-stage mass spectrometry involving consecutive fragmentations) data in positive mode, and full-scan but non-fragmentation spectra in both ionization modes [14, 22, 24, 25]. The present work provides the first high-resolution tandem mass spectrometry (HRMS/MS) fragmentation data for As-Sugars in both ionization modes, offering significantly improved mass accuracy and clearer spectral profiles. These advances enable more reliable structural elucidation and overcome the limitation of earlier low-resolution datasets.

While both ionization modes provided useful information, the positive mode proved practical advantages for structural confirmation, as all four As-Sugars ionized efficiently and produced characteristic fragment ions, especially the diagnostic ion at *m/z* 237.0103 assigned to [C_7_H_14_AsO_4_]^+^. These features improve qualitative identification but do not confer quantitative capability, which remains limited in ESI-Ms due to ion-suppression effects and the absence of suitable standards.

### Ion mobility—mass spectrometry

In this study, ion mobility has been combined with high-resolution mass spectrometry to enhance confidence in the identification of As-Sugars. For the first time, the CCS values were determined for these compounds. CCS is a highly reproducible gas-phase property that is independent of chromatographic conditions and less affected by matrix effects than retention time. CCS values also show good inter-platform reproducibility when standardized calibration is used. Although CCS alone is not sufficiently specific to enable unequivocal molecular identification, in this study it is used as a robust orthogonal parameter that strengthens qualitative identification when combined with accurate-mass and MS/MS data, thereby reinforcing the confirmation of As-Sugars in complex samples.

CCS values were established using extracts of Fucus and Nori (“Sample preparation” section) under optimized IMS conditions. Measurements were performed in triplicate, with mobile phase blanks included to ensure the data reliability. The method showed excellent repeatability, with CCS RSDs below 0.1%. Table 2 summarizes the DT and corresponding CCS values for each As-Sugar.

**Table 2:** Drift times (DT) and CCS values (Å^2^) for the As-sugars studied (*n* = 3)

	**[M+H]**^**+**^		**[M-H]**^**-**^	
As-Sug	DT (ms)	CCS (Å^2^) (RSD %)	DT (ms)	CCS (Å^2^) (RSD %)
Gly-Sug	21.80	161.8 (0.04)	-	
PO_4_-Sug	26.89	197.0 (0.05)	23.30	191.6 (0.08)
SO_3_-Sug	23.64	174.2 (0.09)	23.17	169.8 (0.06)
SO_4_-Sug	23.61	173.7 (0.05)	23.40	171.3 (0.03)

Overall, [M+H]^+^ ions showed larger CCS values than [M-H]^-^ ions, indicating more extended gas-phase conformation upon protonation. Gly-Sug, detected only in positive mode, had the lowest CCS value, consistent with its smaller size and simpler structure. In contrast, PO_4_-Sug showed the highest CCS value in both ionization modes, likely reflecting the increased size and rigidity imparted by the phosphate group. Regarding SO_3_-Sug and SO_4_-Sug, their ions exhibited nearly identical CCS values in both ionization modes, which is consistent with their highly similar structures and resulting comparable gas-phase conformations.

### Sample analysis

Twelve algae samples (six brown, one red, and five green) were processed (in triplicate) following the “Sample preparation” section [7], with minor adjustments to ensure compatibility with UHPLC-Q-Orbitrap, IMS-Q-TOF, the HILIC-Z column, and the HPLC-ICP-MS under IEX conditions. Quantification was performed exclusively by HPLC-ICP-MS under IEX conditions, whereas structural identification was carried out independently using UHPLC-ESI-HRMS and IMS-MS on a HILIC-Z column. Because the chromatographic modes differ completely, retention times from UHPLC-ESI-HRMS were not used to identify the HPLC-ICP-MS peaks; instead, the previously reported information on well-known algae extracts [7] was used to identify As-Sugars retention times in HPLC-ICP-MS.

For UHPLC-Q-Orbitrap and IMS-Q-TOF, response factors were estimated by correlating HPLC-ESI-HRMS peak areas with concentrations determined independently by HPLC-ICP-MS. Substantial variability in response factors was observed among algae samples, reflecting pronounced matrix effects that are well known in ESI-MS quantitation. The variation ranged from 14 to 92%, depending on the As-Sugar and sample, and tended to increase for later-eluting compounds. In positive mode, response factors variability in samples analysed by triplicate reached 43% for Gly-Sug (n_samples_ = 11), 69% for SO_3_-Sug (n_samples_ = 7), 14% for SO_4_-Sug (n_samples_ = 2), and 92% for PO_4_-Sug (n_samples_ = 10). As expected, the earliest-eluting species (SO_4_-Sug) showed poor detectability in many samples, likely due to suppression by co-eluting non-retained matrix components. These results highlight the need for matrix-matched calibration or standard addition approaches when attempting quantitative analysis by HPLC-ESI-HRMS.

The UHPLC-ESI-HRMS screening focused primarily on As-Sugars but extracted-ion traces were also evaluated to explore the presence of other arsenic species. Table 3 shows that As-Sugars were detected as predominant arsenic species across the twelve samples. PO_4_-Sug, SO_3_-Sug, and SO_4_-Sug were also detected in both ionization modes, while Gly-Sug appeared in all samples but only in positive mode. PO_4_-Sug and Gly-Sug were the only As-Sugars detected in red and green algae. PO_4_-Sug was particularly abundant in Nori, while SO_3_-Sug was exclusive to green algae. Notably, SO_4_-Sug was found only in a few samples, confirming its low natural abundance. In the additional extracted-ion traces, we detect dimethylarsinic acid (DMA), which appeared mainly in positive mode, and arsenobetaine (AB), which was observed exclusively in positive mode. In contrast, inorganic arsenic species and monomethylarsonic acid (MMA) were not expected to ionize efficiently under HILIC-ESI conditions; therefore, their absence in the HRMS data does not imply their absence in the samples. Their presence was assessed exclusively by HPLC-ICP-MS.

**Table 3:**
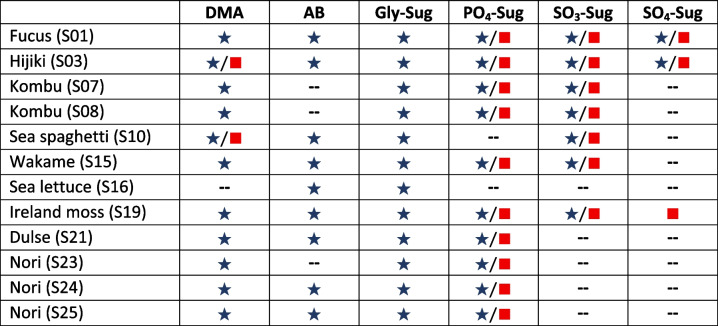
Arsenic species identified in each sample using positive (★) and negative (◼) ionization modes

Using the IMS-Q-TOF system, CCS values were determined for [M+H]^+^ and [M-H]^-^ ions of the As-Sugars detected in the samples. Because Gly-Sug was observed only in positive ion mode, a CCS value was obtained exclusively for its [M+H]^+^ ion (Tables 4 and 5). All CCS values reported correspond to the averaged results from the 72 experiments (12 samples × 3 replicates × 2 extraction volumes). CCS values were highly consistent across samples and replicates, with RSDs below 0.3%, demonstrating excellent intra-day and inter-day precision. Experimental CCS values differed from predicted values by more than 5% (Table 5), likely because existing prediction databases lack structurally related As-Sugar references. This highlights the scarcity of experimental CCS data for As-Sugars and the need for empirical values to refine predictive models.

**Table 4:** Average CCS values (Å^2^) for arsenosugars. Derived values from the mean of all samples and their replicate measurements (*n* = 72)

**As-Sug**	**[M+H]**^**+**^		**[M-H]**^**-**^	
	**CCS (Å**^**2**^**)**	**RSD (%)**	**CCS (Å**^**2**^**)**	**RSD (%)**
Gly-Sug	161.8	0.1	-	
PO_4_-Sug	197.1	0.1	191.6	0.1
SO_3_-Sug	174.1	0.2	170.1	0.3
SO_4_-Sug	173.8	0.2	171.4	0.1

**Table 5:** Average CCS values (Å^2^) for arsenosugars. Predicted values from databases, along with their relative differences (%) compared to experimental measurements

**[M+H]**^**+**^						
**As-Sug**	^**1**^**CCS (Å**^**2**^**)**	**Diff. (%)**	^**2**^**CCS (Å**^**2**^**)**	**Diff. (%)**	^**3**^**CCS (Å**^**2**^**)**	**Diff. (%)**
Gly-Sug	174.5	7.8	162.7	0.5	168.1	3.9
PO_4_-Sug	197.3	0.1	189.2	4.0	-	-
SO_3_-Sug	189.1	8.6	169.1	2.9	182.6	4.9
SO_4_-Sug	190.7	9.7	170.0	2.2	184.8	6.3
**[M-H]**^**-**^						
**As-Sug**	^**1**^**CCS (Å**^**2**^**)**	**Diff. (%)**	^**2**^**CCS (Å**^**2**^**)**	**Diff. (%)**	^**3**^**CCS (Å**^**2**^**)**	**Diff. (%)**
PO_4_-Sug	194.7	1.6	186.8	2.5	-	-
SO_3_-Sug	186.3	9.5	166.9	1.9	182.5	7,3
SO_4_-Sug	187.9	9.6	167.7	2.2	183.8	7,2

^**1**^**CCS**: http://ccsbase.net

^**2**^**CCS**: http://ccs.on-demand.waters.com

^**3**^**CCS**: http://allccs.zhulab.cn

As expected, the CCS values obtained for As-Sugars in this study show good reproducibility under consistent instrument conditions and are largely unaffected by chromatographic or matrix effects. Although slight CCS variation may occur between different IMS platforms due to differences in calibration and operating parameters, CCS remains a robust orthogonal descriptor that complements accurate-mass and MS/MS data for reliable identification and confirmation of As-Sugars in complex algae matrices. This is particularly valuable in HILIC-MS analyses, where retention time shifts and matrix-dependent ionization effects are common. The stability of CCS therefore provides a powerful additional metric that strengthens the overall confidence in As-Sugars identification.

## Conclusions

The UHPLC-HRMS method developed in this study enabled the identification and structural characterization of four major As-Sugars in algae (Gly-Sug, PO_4_-Sug, SO_3_-Sug, and SO_4_-Sug). Use of the HILIC-Z column allowed their simultaneous chromatographic separation, and AIF spectra acquired with the IMS-Q-TOF system provided consistent diagnostic fragmentation patterns that supported confident identification.

Importantly, IMS-Q-TOF enabled the first determination of CCS values for As-Sugars. The CCS values were highly reproducible across samples and independent of matrix composition, establishing CCS as a robust molecular identifier complementary to retention time and MS/MS data for As-Sugar.

While HRMS proved highly effective for qualitative analysis, significant matrix effects limited its suitability for accurate quantification. These results highlight HRMS as a powerful complementary technique to HPLC-ICP-MS, which remains essential for reliable quantitation in complex biological matrices.

## Supplementary Information

Below is the link to the electronic supplementary material.

Supplementary file1 (PDF 254 KB)

## Data Availability

All data generated or analysed during this study are included in this article and its supplementary materials.
