# Supplementary material for: Comprehensive profiling of arsenosugars in algae using UHPLC-HRMS and UHPLC-IMS-Q-TOF
Source: Anal Bioanal Chem. 2026 Mar 27;418(11):3423–33. doi: 10.1007/s00216-026-06454-w (PMC13197290; doi:10.1007/s00216-026-06454-w)
Supplement: Supplementary file 1 — Supplementary file1 (PDF 254 KB) [file 216_2026_6454_MOESM1_ESM.pdf]

SUPPLEMENTARY MATERIAL

COMPREHENSIVE PROFILING OF ARSENOSUGARS IN ALGAE USING UHPLC-HRMS AND UHPLC-IMS-Q-TOF

Alba Morales-Rodríguez[1,2,3], Àngels Sahuquillo[1,4], José Fermín López-Sánchez[1,4], Dolores Barrón[2,3], Encarnación Moyano[1,4]

[1]Departament d'Enginyeria Química i Química Analítica, Universitat de Barcelona, Martí i Franquès, 1-11, 08028, Barcelona, Spain
[2]Departament de Nutrició, Ciències de l'Alimentació i Gastronomia, Campus de l'Alimentació de Torribera, Universitat de Barcelona, Avda. Prat de la Riba, 171, 08921, Sta. Coloma de Gramenet, Barcelona, Spain
[3]Institut de Recerca en Nutrició i Seguretat Alimentària. Universitat de Barcelona (INSA-UB, Recognized as a Maria de Maeztu Unit of Excellence grant (CEX2021-001234-M)), Spain
[4] Institut de Recerca de l'Aigua. Universitat de Barcelona (IdRA-UB), Spain

Table S1: Chromatographic conditions used for the separation of arsenic species.

Table S2: Assignment of fragment ions based on the proposed fragmentation pathways of As-sugars.

Figure S1: All-Ion-Fragmentation (AIF) mass spectra obtained in positive ionization mode using the IMS-Q-TOF system for Gly-Sug.

Table S1. Chromatographic conditions used for the separation of arsenic species.

	**Anionic exchange chromatography**	**Cationic exchange chromatography**
**LC**	Quaternary pump, Agilent 1200 equipped with an autosampler	Quaternary pump, Agilent 1200 equipped with an autosampler
**Column**	Hamilton PRP-X100 (250 mm x 4.1 mm i.d.; 10 µm)	Zorbax 300-SCX (250 mm x 4.6 mm i.d.; 5 µm)
**Precolumn**	Hamilton PRP-X100 (20 mm x 2.0 mm i.d.; 10 µm)	Zorbax 300-SCX (12.5 mm x 4.6 mm i.d.; 5 µm)
**Mobile phase**	A: 30 mM $NH_4H_2PO_4$ pH=5.8 (adjusted with aqueous ammonia)<br>B: 30 mM $NH_4H_2PO_4$ pH=8 (adjusted with aqueous ammonia)	20 mM pyridine pH=2.6 (adjusted with formic acid)
**Flow rate ($mL \cdot min^{-1}$)**	1.5	1.5
**Injection volume (µL)**	100	100
**Pressure (bar)**	160	170
**Arsenic species**	As(III), As(V), MMA, DMA, PO_4-Sug, SO_3-Sug, SO_4-Sug	AB, AC, TMAO, Gly-Sug

Table S2. Assignment of fragment ions based on the proposed fragmentation pathways of As-sugars.

	**Precursor ion**		**Product ion**	
**As-Sug**	***m/z*** **(ppm)**	**Ion assignment**	***m/z*** **(ppm)**	**Ion assignment**
Gly-Sug	329.0574 (-0.6)	$[M+H]^+$	311.0470 (0.1)	$[M+H-H_2O]^+$
			237.0102 (-0.4)	$[C_7H_{14}AsO_4]^+$
			218.9996 (-0.3)	$[C_7H_{12}AsO_3]^+$
			194.9997 (-0.2)	$[C_5H_{12}AsO_3]^+$
			164.9891 (0.2)	$[C_4H_{10}AsO_2]^+$
PO_4-Sug	483.0604 (-0.7)	$[M+H]^+$	465.0500 (-0.1)	$[M+H-H_2O]^+$
			391.0134 (0.0)	$[M+H-C_3H_8O_3]^+$
			329.0577 (0.4)	$[M+H-C_3H_7O_5P]^+$
			237.0101 (-0.7)	$[C_7H_{14}AsO_4]^+$
	481.0444 (-3.7)	$[M-H]^-$	407.0067 (-6.6)	$[M-H-C_3H_6O_2]^-$
			388.9966 (-5.7)	$[M-H-C_3H_8O_3]^-$
			245.0422 (-4.1)	$[M-H-C_7H_{13}AsO_4]^-$
			171.0054 (-5.8)	$[M-H-C_{10}H_{19}AsO_6]^-$
			152.9949 (-6,1)	$[M-H-C_{10}H_{21}AsO_7]^-$
SO_3-Sug	393.0195 (0.0)	$[M+H]^+$	375.0084 (-1.2)	$[M+H-H_2O]^+$
			295.0525 (1.4)	$[M+H-SO_4H_2]^+$
			237.0102 (-0.4)	$[C_7H_{14}AsO_4]^+$
			218.9994 (-1.5)	$[C_7H_{12}AsO_3]^+$
			194.9997 (-0.2)	$[C_5H_{12}AsO_3]^+$
	391.0041 (-2.1)	$[M-H]^-$	372.9919 (-6.3)	$[M-H-H_2O]$-
			269.0321 (-5.7)	$[M-H-C_2H_7AsO]^-$
			155.0011 (-4.9)	$[M-H-C_7H_{13}AsO_4]^-$
			136.9908 (-4.4)	$[M-H-C_7H_{15}AsO_5]^-$
SO_4-Sug	409.0145 (0.2)	$[M+H]^+$	329.0577 (0.4)	$[M+H-SO_3]^+$
			311.0470 (0.0)	$[M+H-SO_4H_2]^+$
			237.0102 (-0.3)	$[C_7H_{14}AsO_4]^+$
			194.9997 (0.2)	$[C_5H_{12}AsO_3]^+$
	406.9988 (-2.7)	$[M-H]^-$	388.9875 (-4.5)	$[M-H-H_2O]$-
			285.0278 (-4.1)	$[M-H-C_2H_7AsO]^-$
			170.9964 (-4.5)	$[M-H-C_7H_{13}AsO_4]^-$
			152.9850 (-8.3)	$[M-H-C_7H_{15}AsO_5]^-$

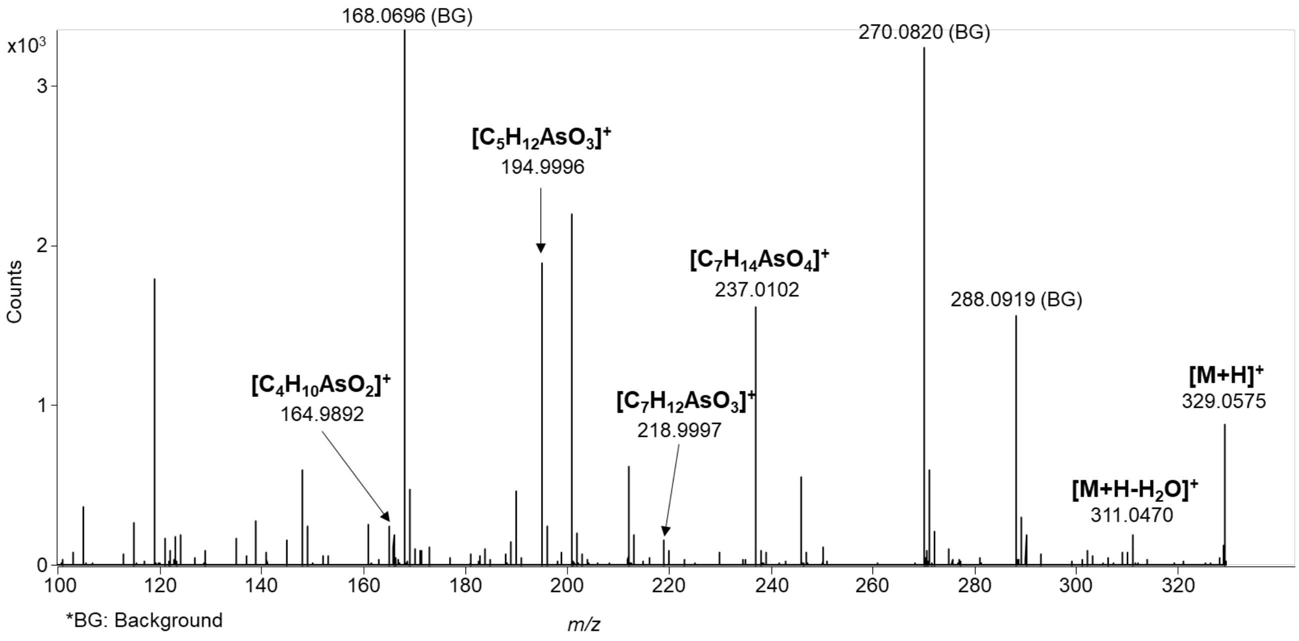

Fig. S1. All-Ion-Fragmentation (AIF) mass spectra obtained in positive ionization mode using the IMS-Q-TOF system for Gly-Sug.
